# Dissociation Between Clinical and Ultrasonographic Response After Radial Shock Wave Therapy in Refractory Plantar Fasciitis

**DOI:** 10.3390/jcm15083068

**Published:** 2026-04-17

**Authors:** Manuel Novo Rigueiro, Fabio Pires Pereira, Ignacio Lete Achirica, Antonio Gómez Caamaño, Francisco Javier Rodríguez Rigueiro, Jesús Rodríguez Figueroa, Arturo González Quintela, Ignacio Novo Veleiro

**Affiliations:** 1 Department of Physical Medicine and Rehabilitation, Álvaro Cunqueiro University Hospital, Estrada de Clara Campoamor, 341, 36213 Vigo, Spain; 2 Department of Radiation Oncology, Clinical University Hospital of Santiago de Compostela, Choupana, 15706 Santiago de Compostela, Spain; ppires@saludcastillayleon.es (F.P.P.); antonio.gomez.caamano@sergas.es (A.G.C.); 3 Department of Radiology, Clinical University Hospital of Santiago de Compostela, Choupana, 15706 Santiago de Compostela, Spain; ignacio.lete.achirica@sergas.es; 4 Department of Engineering and Advanced Technologies, University of Santiago de Compostela (USC), Choupana, 15706 Santiago de Compostela, Spain; fj.rodriguez.rigueiro@usc.es; 5 Department of Physical Medicine and Rehabilitation, Clinical University Hospital of Santiago de Compostela, Choupana, 15706 Santiago de Compostela, Spain; jesus.rodriguez.figueroa@sergas.es; 6 Department of Internal Medicine, Clinical University Hospital of Santiago de Compostela, Choupana, 15706 Santiago de Compostela, Spain; arturo.gonzalez.quintela@usc.es (A.G.Q.); ignacio.novo.veleiro@usc.es (I.N.V.)

**Keywords:** heel pain, treatment outcome, ultrasonography, prognosis

## Abstract

**Background**: Chronic plantar fasciitis refractory to conservative treatment is a frequent cause of persistent heel pain and functional limitation. Although radial extracorporeal shock wave therapy (rESWT) has shown potential benefit, the relationship between clinical improvement and structural ultrasonographic changes remains unclear. The aim of this study was to evaluate the clinical, functional, and ultrasonographic outcomes associated with rESWT in patients with refractory plantar fasciitis. **Methods**: We conducted a prospective observational single-center study including 287 patients with plantar fasciitis refractory to conservative treatment for at least 6 months and confirmed by ultrasonography (plantar fascia thickness >4 mm). All patients received four weekly sessions of rESWT. Pain intensity (visual analog scale [VAS]), foot function (Foot Function Index [FFI]), quality of life (EQ-5D), and plantar fascia thickness were assessed at baseline and 3 months after treatment. **Results**: Significant improvements were observed in pain (mean VAS change, −3.73 points), function (mean FFI-disability change, −32.37 points), and quality of life (improvement in at least one EQ-5D dimension in 81.5% of patients) (all *p* < 0.001). The mean reduction in plantar fascia thickness was 0.14 mm. Most responders (71.8%) showed clinical improvement despite the absence of a relevant structural change, defined as a reduction in plantar fascia thickness <0.5 mm. In multivariate analysis, physically demanding occupations were associated with a lower probability of response (odds ratio, 0.32; 95% confidence interval, 0.17–0.63). The prognostic model showed moderate discrimination (area under the curve, 0.71). **Conclusions**: In this observational cohort, rESWT was associated with improvements in pain, function, and quality of life in patients with refractory plantar fasciitis. Clinical improvement frequently occurred despite minimal changes in plantar fascia thickness, suggesting that ultrasonographic thickness may not adequately reflect symptomatic evolution. However, the absence of a control group prevents causal interpretation of these findings.

## 1. Introduction

Plantar fasciitis is one of the most common causes of heel pain in adults and represents a frequent reason for consultation in musculoskeletal practice. It has been estimated that up to 10% of the population may experience plantar heel pain during their lifetime, and a relevant proportion of patients develop persistent symptoms associated with pain, functional limitation, and impaired quality of life [1,2,3].

Conservative treatment is usually considered the first-line approach and commonly includes activity modification, stretching exercises, physical therapy, foot orthoses, and nonsteroidal anti-inflammatory drugs [3]. Corticosteroid injections may provide short-term pain relief, but their benefits tend to decrease over time and repeated injections have been associated with potential complications, including plantar fascia rupture and fat pad atrophy [4]. Other interventions, such as platelet-rich plasma, have also been explored, although current evidence remains heterogeneous and conclusions remain inconsistent [5,6].

Extracorporeal shock wave therapy (ESWT) has emerged as a minimally invasive treatment option for chronic and refractory plantar fasciitis. Several randomized controlled trials and meta-analyses have reported improvements in pain and function after both focused and radial ESWT [7,8,9]. However, the available evidence remains heterogeneous, with differences in study design, treatment protocols, and outcome definitions across studies [7,8,9,10,11,12,13]. Radial ESWT has gained wide acceptance in rehabilitation settings because of its favorable safety profile, ease of application, and reproducibility.

Another unresolved issue concerns the relationship between symptomatic improvement and structural changes in the plantar fascia. Ultrasonographic assessment of plantar fascia thickness is commonly used for diagnosis and follow-up, but its value as a biomarker of clinical evolution remains uncertain. Some studies have reported reductions in fascial thickness after ESWT, whereas others have found weak or no correlation between ultrasonographic findings and patient-reported improvement [10,11,12,13].

Large prospective cohorts reflecting routine clinical practice remain limited, particularly those simultaneously evaluating pain, function, quality of life, and ultrasonographic findings. Therefore, the aim of the present study was to evaluate the clinical, functional, and ultrasonographic outcomes of radial ESWT in patients with refractory plantar fasciitis, with particular attention to the possible dissociation between symptomatic improvement and structural ultrasonographic changes. A secondary objective was to explore baseline factors associated with treatment response in routine rehabilitation practice.

## 2. Materials and Methods

### 2.1. Study Design and Ethical Approval

A prospective observational single-center study was conducted at the University Hospital Complex of Santiago de Compostela and within the Santiago–Barbanza health area between January 2019 and December 2023. The study protocol was approved by the Galician Research Ethics Committee (approval code 2021/120, approved on 19 October 2021). All participants provided written informed consent prior to inclusion, in accordance with the Declaration of Helsinki and European biomedical research regulations.

### 2.2. Study Population

Consecutive adult patients referred from Primary Care and other hospital departments for persistent plantar heel pain compatible with plantar fasciitis were screened for eligibility. A total of 309 patients were assessed for eligibility during the study period.

Inclusion criteria were:

Age ≥ 18 years.

Plantar heel pain lasting at least 6 months and refractory to conservative treatment.

Diagnosis of plantar fasciitis confirmed by high-resolution ultrasound, defined as proximal plantar fascia thickness > 4 mm. 

Ultrasonographic assessment performed using a Canon Aplio i800 system with a multifrequency 5–18 MHz linear probe (Canon Medical Systems Corporation, Otawara, Tochigi, Japan), conducted in all cases by the same specialist physician.

Plain radiographs were performed when clinically indicated, particularly in patients with atypical symptoms or when alternative diagnoses were suspected, but they were not obtained systematically in all participants.

Exclusion criteria were:

Concomitant conditions potentially explaining heel pain, including Baxter neuropathy, stress fractures, inflammatory arthropathy, lumbar radiculopathy, or uncontrolled adjacent tendinopathies.

Pregnancy, pacemaker carriers, or other contraindications to radial extracorporeal shock wave therapy.

Comorbid conditions such as fibromyalgia or rheumatologic diseases were not considered exclusion criteria when plantar fasciitis remained the main clinical diagnosis, because the aim of the study was to reflect real-world clinical practice in a rehabilitation setting.

### 2.3. Previous Conservative Treatments

Before inclusion, patients had received conservative treatment prescribed by rehabilitation physicians or orthopaedic surgeons, following standard guidelines for plantar fasciitis. These interventions were delivered outside the research protocol and included stretching and strengthening exercises for the plantar fascia and triceps surae, foot orthoses, conventional physiotherapy, local cryotherapy, and topical or oral nonsteroidal anti-inflammatory drugs [3]. In some cases, patients had also previously received local corticosteroid injections, which were recorded from the clinical history.

During the treatment period and follow-up, patients were allowed to continue their usual conservative measures and analgesic medication as needed, according to routine clinical practice. However, the type, dose, and frequency of concomitant medication and conservative measures were not systematically recorded. A formal patient diary was not used during follow-up because the study was designed to reflect routine clinical practice rather than a controlled interventional protocol.

### 2.4. Shock Wave Intervention

After verification of eligibility criteria and written informed consent during the first visit, patients underwent radial extracorporeal shock wave therapy using the BTI Masterpuls^®^ MP100 device (BTI Biotechnology Institute, Vitoria-Gasteiz, Spain). All treatment sessions were performed by the same specialist physician under direct medical supervision.

The treatment protocol consisted of four weekly sessions with the following parameters:

Pressure: 3 bars.

Impulses per session: 2000.

Frequency: 10 Hz.

This protocol was selected in accordance with the recommendations of the European Society for Musculoskeletal Shockwave Therapy and previously published evidence on the use of radial shock wave therapy for chronic and/or refractory plantar fasciitis [12]. In patients with bilateral plantar fasciitis, only the most symptomatic foot was considered for analysis.

### 2.5. Clinical and Functional Assessment

All patients were assessed at baseline (before the first treatment session) and at 3 months after completion of the shock wave protocol. All evaluations were performed by the same physician to ensure interobserver consistency. The ultrasonographic evaluator was not formally blinded to the intervention, and intra-rater reliability was not specifically assessed.

The standardized assessment included:

- **Pain intensity**, assessed using the Visual Analog Scale (VAS; range, 0–10) [13].
- **Foot function**, evaluated with the Foot Function Index (FFI), including pain, disability, and activity limitation subscales, as well as the total score [14].
- **Health-related quality of life**, assessed using the EuroQol-5D (EQ-5D), which evaluates mobility, self-care, usual activities, pain/discomfort, and anxiety/depression [15].
- **Ultrasonographic assessment**, consisting of measurement of proximal plantar fascia thickness at the calcaneal insertion, expressed in millimeters [16].

Demographic variables, laterality of involvement, occupational physical demands, relevant comorbidities (diabetes mellitus, hypertension, dyslipidemia, hypothyroidism, fibromyalgia, and rheumatologic history), and lifestyle habits (smoking and alcohol consumption) were also recorded.

### 2.6. Definition of Treatment Response

The primary outcome was improvement in pain at 3 months, assessed by change in the VAS score.

A favorable pain response was defined as a final VAS score < 5 points or a reduction of ≥2 points from baseline, in accordance with previously established thresholds for clinically meaningful improvement [6].

Secondary response criteria included:

- A reduction of ≥7 points in the total FFI score, corresponding to the minimum clinically important difference reported in the original validation study [14].
- Improvement in health-related quality of life, defined as a reduction of ≥1 level in at least one EQ-5D dimension or an increase of ≥0.05 in the EQ-5D index [15].
- Ultrasonographic reduction in proximal plantar fascia thickness ≥0.5 mm from baseline [16].
- Clinical discharge, defined as a final VAS score < 5 points with acceptable functional recovery in daily activities.

Patients were classified as responders if they met the primary criterion or, alternatively, at least two of the secondary criteria.

### 2.7. Statistical Analysis

Statistical analyses were performed using IBM SPSS Statistics version 29.0 (IBM Corp., Armonk, NY, USA). Continuous variables were expressed as mean ± standard deviation or median (interquartile range), depending on their distribution, assessed using the Shapiro–Wilk test.

Pre- and post-treatment comparisons were performed using paired Student *t* tests for normally distributed continuous variables and Wilcoxon signed-rank tests for non-normally distributed variables, according to the results of the Shapiro–Wilk test. For consistency of presentation, continuous variables are displayed in the tables as mean ± standard deviation, although non-parametric tests were applied when the distributional assumptions for parametric testing were not met. The absolute differences, 95% confidence intervals, and standardized effect sizes (Cohen’s dz) were calculated. Given the multiple comparisons performed, the results should be interpreted with caution because the analyses were not adjusted for multiplicity.

Associations between ultrasonographic changes and clinical improvement were assessed using Pearson or Spearman correlation coefficients, depending on data distribution. Subgroup analyses were performed according to sex, age, comorbidities, and occupational physical demands.

A multivariate logistic regression model was constructed to identify independent prognostic factors associated with clinical outcome. The final multivariate model was exploratory and included clinically relevant baseline variables selected a priori; therefore, the results should be interpreted cautiously given the limited number of non-responders in relation to the number of candidate predictors. Model discrimination was assessed using receiver operating characteristic curve analysis, with calculation of the area under the curve. A two-sided *p* value < 0.05 was considered statistically significant.

The reporting of this study conforms to the Strengthening the Reporting of Observational Studies in Epidemiology (STROBE) guidelines.

## 3. Results

### 3.1. Study Flow and Baseline Characteristics

**Figure 1:**
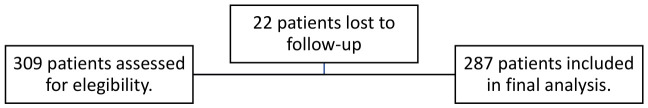
Patient flow diagram. Of the 309 patients assessed for eligibility, 22 were lost to follow-up and were not included in the final analysis. A total of 287 patients completed the radial shock wave therapy protocol and were included in the study.

Of the 309 patients assessed for eligibility, 22 were lost to follow-up and therefore not included in the final analysis. The final cohort consisted of 287 patients who completed the four-session radial extracorporeal shock wave therapy protocol and were included in the analysis (Figure 1).

The mean age of the cohort was 54.3 ± 8.9 years, 178 of whom (62.0%) were female. Involvement was predominantly right-sided (54.7%), followed by left-sided (34.8%) and bilateral (10.5%). A majority of patients (54.0%) reported occupations involving significant physical demands.

The most prevalent comorbidities found were dyslipidemia (32.1%), arterial hypertension (27.9%), and diabetes mellitus (12.2%). Hypothyroidism was present in a 9.8% of patients, and fibromyalgia in 4.5%. Regarding lifestyle factors, a 20.6% of participants were smokers and 12.9% reported regular alcohol consumption. Previous surgery on the affected foot had been performed in 5.6% of cases, and 19.9% of patients had received corticosteroid injections prior to shock wave therapy (Table 1).

### 3.2. Clinical, Functional, and Ultrasonographic Outcomes

At 3 months after completion of radial extracorporeal shock wave therapy, statistically significant improvements were observed in all clinical and functional outcome measures (Table 2).

Pain intensity measured by the Visual Analog Scale decreased from a baseline mean of 7.62 ± 1.04 to 3.90 ± 2.77, corresponding to a mean reduction of 3.73 points (95% confidence interval [CI], −4.00 to −3.45; *p* < 0.001). A clear improvement was found in all subscales of the Foot Function Index, pointing to a clear improvement with mean reductions of 37.93 points in the pain domain, 32.37 points in the disability domain, and 15.37 points in activity limitation (all *p* < 0.001).

Health-related quality of life also improved significantly, with reductions observed across all EQ-5D dimensions, particularly in mobility, usual activities, and pain/discomfort (all *p* < 0.001).

In contrast, modest results were found for ultrasonographic plantar fascia thickness. Thus, mean thickness decreased from 5.76 ± 0.99 mm at baseline to 5.62 ± 1.03 mm at follow-up, corresponding to a mean reduction of 0.14 mm (95% CI, −0.16 to −0.11; *p* < 0.001).

No adverse events or treatment-related complications were reported during the intervention or follow-up period.

### 3.3. Treatment Response Rates

**Figure 2:**
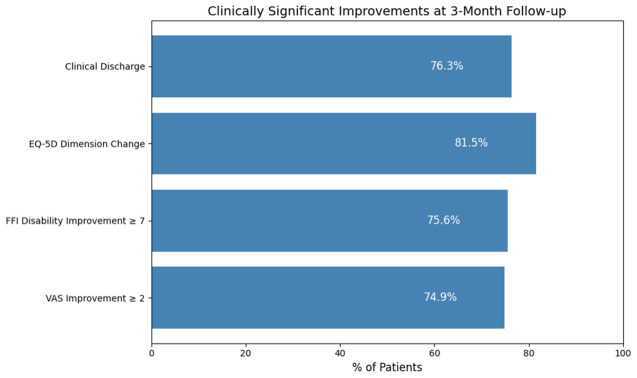
Percentage of patients meeting clinically significant improvement criteria at 3 months following radial extracorporeal shock wave therapy.

Based on the predefined criteria, 219 of the 287 patients (76.3%) were classified as responders (Figure 2).

Specifically:

- A reduction in VAS pain score of ≥2 points was observed in 215 patients (74.9%).
- A final VAS score < 5 points at 3 months was achieved by 209 patients (72.8%).
- A clinically meaningful improvement of ≥7 points in the FFI disability subscale was observed in 75.6% of patients.
- Improvement in at least one EQ-5D dimension occurred in 81.5% of patients.
- Clinical discharge criteria were met by 219 patients (76.3%).

Ultrasonographic reduction in plantar fascia thickness ≥0.5 mm was observed in only 17 patients (5.9%), and a reduction ≥10% was observed in 10 patients (3.5%).

### 3.4. Relationship Between Clinical and Ultrasonographic Outcomes

Most patients demonstrated clinically meaningful improvement in pain, function, or quality of life without corresponding ultrasonographic structural change. Specifically, 206 patients (71.8%) showed clinical improvement in the absence of a plantar fascia thickness reduction ≥0.5 mm.

**Figure 3:**
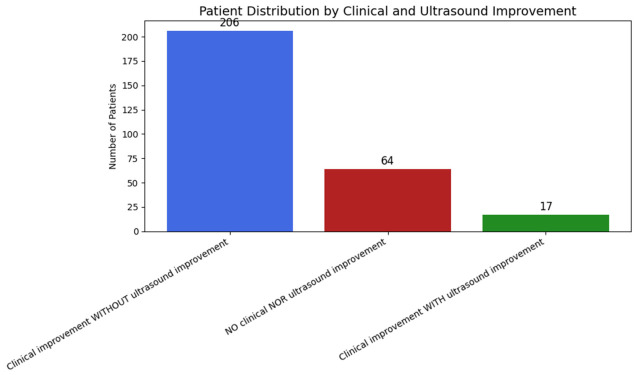
Distribution of patients according to the presence or absence of clinical and ultrasound improvement three months after treatment.

Only 17 patients (5.9%) showed both clinical improvement and relevant ultrasonographic thickness reduction, whereas 64 patients (22.3%) showed neither clinical nor structural improvement. The distribution of patients according to clinical and ultrasonographic response is shown in Figure 3.

No significant correlations were observed between the magnitude of change in plantar fascia thickness and changes in pain or functional outcomes.

### 3.5. Comparison Between Responders and Non-Responders

**Figure 4:**
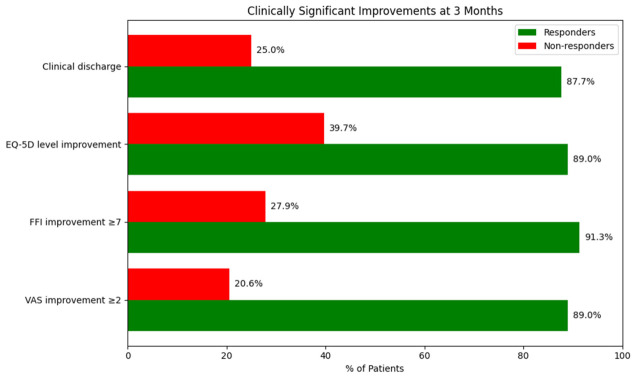
Percentage of patients who achieved clinically significant improvement at 3 months, according to responder and non-responder status.

We assessed the percentage of patients who achieved clinically significant improvements in each of the secondary criteria (reduction of ≥2 points in VAS, ≥7 points in FFI-disability, level change in EQ-5D, and clinical discharge) (Figure 4).

Responders demonstrated significantly better baseline clinical and functional status compared with nonresponders. Baseline VAS pain scores, all FFI subscale scores, plantar fascia thickness, and all EQ-5D dimension scores were significantly lower in responders than in nonresponders (all *p* < 0.001) (Table 3). These baseline differences should be interpreted cautiously, as they may have influenced the probability of achieving the predefined response thresholds and may partly reflect regression-to-the-mean effects.

With respect to baseline demographic and clinical characteristics (Table 4), the proportion of patients with physically demanding occupations was significantly higher among nonresponders than responders (76.5% vs. 50.7%; *p* = 0.0003). Bilateral plantar fasciitis was more frequent among responders (14.2% vs. 4.4%; *p* = 0.031). No significant differences were observed between groups regarding sex, age, comorbidities, lifestyle factors, previous surgery, or prior corticosteroid injection.

### 3.6. Prognostic Factors Associated with Clinical Outcome

A multivariate logistic regression model was constructed to identify factors associated with outcome (Table 5). After adjustment for demographic, clinical, and lifestyle variables, having a physically demanding occupation was the only factor independently associated with a lower probability of response to radial shock wave therapy (odds ratio, 0.32; 95% CI, 0.17–0.63; *p* = 0.001).

No other variables showed statistically significant associations with treatment response in the adjusted model.

**Figure 5:**
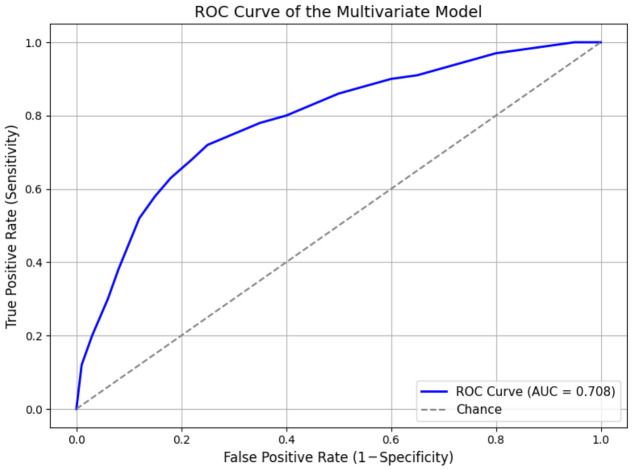
ROC (receiver operating characteristic) curve of the multivariate logistic regression model for prognostic classification of clinical outcome. The area under the curve (AUC) was 0.708, indicating acceptable discriminative ability. The diagonal line represents random classification (AUC = 0.5).

The predictive performance of the model was evaluated using receiver operating characteristic curve analysis. The area under the curve was 0.71, indicating moderate discriminative ability (Figure 5). Using the optimal threshold determined by the Youden index (0.824), the model achieved a sensitivity of 49% and a specificity of 87%, with a positive predictive value of 92% and a negative predictive value of 35% (Table 6).

## 4. Discussion

The present study showed that patients with chronic plantar fasciitis refractory to conservative treatment experienced significant improvements in pain, foot function, and quality of life after radial extracorporeal shock wave therapy. These improvements were accompanied by a reduction in plantar fascia thickness, although the magnitude of structural change was smaller than the clinical improvement observed. The findings therefore support the possibility that symptom relief may occur despite limited ultrasonographic remodeling during the first months after treatment. Although the magnitude of clinical improvement observed in the present cohort was relevant and consistent with previous studies, the observational and uncontrolled design of the study prevents a definitive causal relationship being established between radial extracorporeal shock wave therapy and the observed changes. Part of the improvement may reflect placebo effects, regression to the mean, or the natural course of the disease [6,17]. In addition, patients were allowed to continue their previous conservative measures and analgesic medication during the treatment and follow-up period. Because these concomitant interventions were not systematically quantified, their potential contribution to symptom improvement cannot be excluded and should be considered an additional source of confounding.

The observed magnitude in our cohort for pain reduction (mean VAS decrease of 3.7 points) goes in line with results reported in randomized controlled trials and meta-analyses evaluating both focused and radial shock wave modalities, where reductions of approximately 3 to 4 points have been described at short- to mid-term monitoring [7,8,9]. In a similar way, the improvements reported and discussed in Foot Function Index scores, particularly for the disability domain, indicate a substantial functional benefit extending beyond pain relief alone. The highlighted outcomes are comparable to the results reported in other minimally invasive interventions, including platelet-rich plasma injections. However, direct comparisons are limited by heterogeneity in study designs and outcome definitions [18,19].

The reduction in plantar fascia thickness observed after treatment was statistically significant but modest. Moreover, some patients experienced marked clinical improvement despite minimal or absent structural change on ultrasound. This finding is in agreement with previous reports suggesting that ultrasonographic changes may lag behind clinical evolution and that plantar fascia thickness alone should not be considered a surrogate marker of treatment response. Even if a small but statistically significant reduction in mean fascia thickness was observed at the group level, in a limited number of patients, a thickness reduction was found exceeding 0.5 mm. Thus, a majority of respondents experienced significant improvements in pain and function without relevant changes in plantar fascia thickness on ultrasound. This observation goes in line with previous reports indicating that the symptomatic improvement after shock wave therapy is not necessarily directly correlating with macroscopic structural remodeling being detectable by ultrasound imaging [10,11,20]. Therefore, the present findings seem to support the hypothesis that therapeutic effects of radial extracorporeal shock wave therapy may be mediated through functional, neuromodulatory or microvascular mechanisms rather than through direct structural normalization of the fascia [7,11,21].

The multivariate model identified several baseline variables associated with a lower probability of response, particularly higher baseline pain, worse functional status, and longer symptom duration. However, the predictive performance of the model was only moderate, with an area under the curve of 0.71, and no external validation was performed. Therefore, the model should be interpreted as exploratory and hypothesis-generating rather than as a tool ready for routine clinical decision-making. In the present cohort, having a physically demanding occupation was independently linked to a lower probability of response to the treatment, even after adjustment for demographic and clinical variables. This finding is consistent with previous evidence and studies suggesting that continued mechanical overload may limit the effectiveness of radial extracorporeal shock wave therapy in plantar fasciitis [20]. Conversely, demographic factors and clinical history parameters such as age, gender, metabolic comorbidities and previous corticosteroid injections, were not independently associated with treatment response in the multivariate model, what reinforces the findings reported by different studies on this subject [17,20].

### Study Limitations

The present study has several limitations. First, the absence of a control or sham group represents the main limitation and prevents differentiating the effect of radial extracorporeal shock wave therapy from placebo effects, regression to the mean, or the natural evolution of symptoms. Second, the follow-up period was limited to 3 months, which may be insufficient to assess the durability of clinical improvement, recurrence rates, or long-term structural remodeling, and may also partly explain the dissociation observed between symptomatic improvement and ultrasonographic findings.

Third, previous conservative treatments and analgesic medication were not discontinued and were not systematically quantified during follow-up. Consequently, their possible influence on the observed outcomes cannot be excluded. Fourth, radiographs were not systematically performed in all patients, but only when clinically indicated, and not all potential alternative causes of heel pain may have been completely excluded despite the clinical and ultrasonographic assessment. Fifth, all ultrasonographic evaluations were performed by the same specialist physician, who was not blinded to the intervention, and intra-rater reliability was not formally assessed, which may have introduced observer bias. Finally, the predictive model was internally derived using a relatively high number of candidate variables in relation to the number of non-responders, and no external validation was performed, which limits its generalizability and increases the risk of overfitting.

From a practical perspective, the findings suggest that radial extracorporeal shock wave therapy may be considered as a therapeutic option in patients with chronic plantar fasciitis refractory to standard conservative treatment, particularly when pain and functional impairment remain clinically relevant after at least 6 months of symptoms. Nevertheless, physicians should interpret the expected benefit cautiously and should not rely exclusively on ultrasonographic changes when evaluating treatment response.

## 5. Conclusions

In this observational cohort, radial extracorporeal shock wave therapy was associated with improvements in pain, function, and quality of life in patients with refractory plantar fasciitis. Clinical improvement frequently occurred despite minimal changes in plantar fascia thickness, suggesting that ultrasonographic thickness alone may not adequately reflect symptomatic evolution. Because of the observational and uncontrolled design, these findings should be interpreted cautiously and should not be considered evidence of a causal treatment effect.

## Figures and Tables

**Table 1:** Baseline demographic and clinical characteristics of the study population (n = 287).

Variable	Value
Age (years)	54.3 ± 8.9
Female sex	178 (62.0%)
Male sex	109 (38.0%)
Right-sided involvement	157 (54.7%)
Left-sided involvement	100 (34.8%)
Bilateral involvement	30 (10.5%)
Physically demanding occupation	155 (54.0%)
Fibromyalgia	13 (4.5%)
Rheumatologic history	19 (6.6%)
Diabetes mellitus	35 (12.2%)
Hypothyroidism	28 (9.8%)
Dyslipidemia (elevated cholesterol)	92 (32.1%)
Arterial hypertension	80 (27.9%)
Smoker	59 (20.6%)
Regular alcohol consumption	37 (12.9%)
Prior surgery on the affected foot	16 (5.6%)
Prior corticosteroid injection	57 (19.9%)
Mean municipal slope (%)	5.7 ± 2.8

Values expressed as mean ± SD or n (%).

**Table 2:** Clinical, functional and structural outcomes at 3-month follow-up after radial extracorporeal shock wave therapy.

Variable	Pre (Mean ± SD)	Post (Mean ± SD)	Δ	95% CI	*p*	dz
Fascial thickness (mm)	5.76 ± 0.99	5.62 ± 1.03	−0.14	−0.16–−0.11	<0.001	−0.55
Pain (VAS)	7.62 ± 1.04	3.90 ± 2.77	−3.73	−4.00–−3.45	<0.001	−1.57
FFI–Pain	72.98 ± 10.76	35.05 ± 27.69	−37.93	−40.75–−35.11	<0.001	−1.56
FFI–Disability	66.60 ± 11.94	34.23 ± 25.29	−32.37	−34.93–−29.81	<0.001	−1.47
FFI–Limitation	38.44 ± 7.84	23.06 ± 13.56	−15.37	−16.80–−13.94	<0.001	−1.25
EQ−5D Mobility	2.36 ± 0.57	1.71 ± 0.76	−0.64	−0.72–−0.57	<0.001	−0.94
EQ−5D Self-care	1.45 ± 0.59	1.32 ± 0.52	−0.13	−0.19–−0.06	<0.001	−0.23
EQ−5D Usual activities	2.29 ± 0.60	1.62 ± 0.74	−0.67	−0.76–−0.58	<0.001	−0.88
EQ−5D Pain/discomfort	2.02 ± 0.66	1.51 ± 0.69	−0.51	−0.60–−0.43	<0.001	−0.73
EQ−5D Anxiety/depression	1.84 ± 0.66	1.57 ± 0.75	−0.27	−0.35–−0.18	<0.001	−0.36

Values are expressed as mean ± standard deviation (SD). Δ = absolute difference; 95% CI = 95% confidence interval; *p* = *p*-value; dz = Cohen’s effect size.

**Table 3:** Comparison of baseline clinical, functional, and quality-of-life variables between responders and non-responders to radial shock wave therapy.

Variable	Responders (n = 219)	Non-Responders (n = 68)	*p*-Value
Baseline VAS pain score	7.37 ± 1.01	8.46 ± 0.63	<0.0001
FFI–Pain	70.94 ± 10.80	79.57 ± 7.54	<0.0001
FFI–Disability	64.36 ± 11.42	73.81 ± 10.72	<0.0001
FFI–Activity Limitation	37.08 ± 7.83	42.79 ± 6.14	<0.0001
Baseline fascial thickness (mm)	5.56 ± 0.80	6.39 ± 1.26	<0.0001
EQ-5D Mobility	2.22 ± 0.55	2.78 ± 0.42	<0.0001
EQ-5D Self-care	1.35 ± 0.52	1.76 ± 0.67	<0.0001
EQ-5D Usual activities	2.18 ± 0.58	2.65 ± 0.51	<0.0001
EQ-5D Pain/Discomfort	1.90 ± 0.63	2.40 ± 0.60	<0.0001
EQ-5D Anxiety/Depression	1.73 ± 0.60	2.18 ± 0.71	<0.0001

Values are expressed as mean ± standard deviation. Student’s *t*-test for independent samples was used. Statistically significant differences were considered for *p* < 0.05. Responders showed better initial values in all analyzed scales.

**Table 4:** Comparison of baseline variables between responders and non-responders to radial shock wave therapy.

Variable	Responders (n = 219)	Non-Responders (n = 68)	*p*-Value
Male sex	60 (27.4%)	19 (27.9%)	1.0000
Age > 54 years	113 (51.6%)	31 (45.6%)	0.4672
Physically demanding occupation	111 (50.7%)	52 (76.5%)	0.0003
Municipal average slope > 5.7%	82 (37.4%)	17 (25.0%)	0.0819
Fibromyalgia	39 (17.8%)	6 (8.8%)	0.1120
Rheumatologic history	7 (3.2%)	2 (2.9%)	1.0000
Diabetes mellitus	47 (21.5%)	12 (17.6%)	0.6114
Hypothyroidism	77 (35.2%)	19 (27.9%)	0.3396
Dyslipidemia	91 (41.6%)	19 (27.9%)	0.0609
Hypertension	62 (28.3%)	12 (17.6%)	0.1102
Smoker	31 (14.2%)	11 (16.2%)	0.8293
Alcohol consumption	18 (8.2%)	4 (5.9%)	0.6129
Previous surgery on the affected foot	5 (2.3%)	3 (4.4%)	0.3992
Previous corticosteroid injection	96 (43.8%)	33 (48.5%)	0.5891
Right-sided involvement	100 (45.7%)	29 (42.6%)	0.7664
Left-sided involvement	88 (40.2%)	35 (51.5%)	0.1329
Bilateral involvement	31 (14.2%)	3 (4.4%)	0.0312

Absolute values and percentages are shown. Differences were analyzed using the Chi-square test or Fisher’s exact test, as appropriate. Statistical significance was considered for *p* < 0.05.

**Table 5:** Multivariate logistic regression identifying prognostic factors associated with clinical outcome after radial extracorporeal shock wave therapy.

Variable	OR	95% CI Lower	95% CI Upper	*p*-Value
Male sex	1.00	0.49	2.06	0.998
Age > 54 years	1.02	0.56	1.87	0.943
Physically demanding occupation	0.32	0.17	0.63	0.001
Mean municipal slope > 5.7%	1.63	0.84	3.15	0.147
Fibromyalgia	1.77	0.64	4.88	0.270
Rheumatologic history	0.93	0.16	5.53	0.938
Diabetes mellitus	0.83	0.35	1.96	0.666
Hypothyroidism	1.26	0.61	2.61	0.525
Dyslipidemia (cholesterol)	1.21	0.54	2.72	0.647
Arterial hypertension	1.65	0.69	3.93	0.258
Smoker	0.55	0.21	1.43	0.221
Alcohol consumption	1.93	0.49	7.65	0.350
Prior surgery on the same foot	0.45	0.08	2.48	0.362
Prior corticosteroid infiltration	0.65	0.35	1.20	0.168
Right-sided involvement	0.48	0.15	1.58	0.225
Left-sided involvement	0.37	0.11	1.22	0.103

Odds ratios (OR), 95% confidence intervals (CI), and *p*-values are presented. An OR < 1 indicates a lower likelihood of being a responder. The dependent variable was responder status (yes/no), as defined by the clinical and functional criteria established in the study.

**Table 6:** Diagnostic performance of the multivariate model using the optimal classification threshold (0.824) determined by the Youden index.

Metric	Value
Sensitivity (TPR)	0.49
Specificity (TNR)	0.87
Positive Predictive Value (PPV)	0.92
Negative Predictive Value (NPV)	0.35
Optimal Threshold (Youden Index)	0.824
Area Under Curve (AUC)	0.71

The table presents sensitivity (TPR; true positive rate), specificity (TNR; true negative rate), positive predictive value (PPV), negative predictive value (NPV), and the area under the curve (AUC).

## Data Availability

The data presented in this study are available on request from the corresponding author. The data are not publicly available due to ethical and privacy restrictions.
